# Psychometric properties of the Parenting Stress Index-Short Form in a Peruvian sample

**DOI:** 10.1186/s41155-024-00327-w

**Published:** 2024-09-27

**Authors:** Katlin T. González-López, Sheyra N. Vásquez-Chingay, Raquel A. Rodrigo-Tintaya, Flor V. Leiva-Colos, Wilter C. Morales-García, Cristian E. Adriano-Rengifo

**Affiliations:** 1 Faculty of Health Sciences, Professional School of Psychology, Universidad Peruana Unión, Lima, Perú; 2 Faculty of Health Sciences, Professional School of Human Medicine, Universidad Peruana Unión, Lima, Perú

**Keywords:** Parental stress, PSI-SF, Factor analysis, Validity, Reliability, Invariance

## Abstract

**Background:**

The stress experienced by parents in fulfilling their parental role has consequences for couple dynamics, parent–child interactions, and the mental health of parents. However, studies on the psychometric properties of the PSI-SF, particularly among Latin American parents, are scarce. Therefore, this study aims to evaluate the psychometric properties of the Spanish version of the Parenting Stress Index-Short Form (PSI-SF).

**Methods:**

The participants were Peruvian mothers and fathers with children in early childhood and primary education, with a mean age of 34.4 years (SD = 6.8). The sample was obtained in two phases: 130 participants for the Exploratory Factor Analysis (EFA) and 791 participants for the Confirmatory Factor Analysis (CFA).

**Results:**

The study results show a modified three-factor structure of the PSI-SF scale, with adequate fit indices (GFI = .99, AGFI = .99, SRMR = .024, CFI = .98, TLI = .98, RMSEA = .074) and loadings above 0.40. Additionally, the three factors of the scale demonstrated high reliability, with Cronbach's alpha and McDonald's omega values for Parental Distress (α = .94; ω = .95), Parent–Child Dysfunctional Interaction (α = .97; ω = .97), and Difficult Child (α = .94; ω = .94). The scale was also found to be invariant with respect to gender.

**Conclusion:**

In conclusion, the study results suggest that the modified PSI-SF has adequate psychometric properties and is invariant for assessing parental stress in Peruvian fathers and mothers with children in early childhood and primary education.

## Introduction

Parental stress is defined as the tension and anxiety parents experience due to the demands associated with raising children. This phenomenon has captured the attention of psychological and sociological research over the past decades because of its significant implications for both parents and children (Abidin, 1992; Crnic & Acevedo, 1995; Deater-Deckard, 2004) . Parental stress can have profound impacts on the mental and physical health of parents, as well as on the development and well-being of children (Crnic & Low, 2002). Lazarus and Folkman's (1984) stress and coping model offers a useful theoretical framework for understanding parental stress. This model emphasizes the importance of stress perception and the resources available to cope with it. According to this approach, parents experience stress when they perceive a discrepancy between parental demands and their ability to meet those demands. Furthermore, the literature suggests that parental stress can be influenced by contextual and cultural factors, as well as individual characteristics of parents, such as personality and coping strategies (Belsky, 1984; Rodrigo, 2010).

Parental stress is a widely studied phenomenon in the social and health sciences due to its significant impact on the well-being of both parents and children. Early studies on parental stress focused on identifying sources of stress, such as economic burden, lack of social support, and expectations about the parental role (Cheng & Lai, 2023). Socioeconomic status, children's health conditions, and interpersonal relationships within the household are determining factors of parental stress. Parents of children with special needs, such as those with autism or epilepsy, tend to report higher levels of stress due to the additional care demands and concerns about their children's future (Operto et al., 2023; Papadopoulos et al., 2023). Similarly, parents in low-income settings report higher levels of parental stress due to economic limitations and lack of social support (Nepomnyaschy & Garfinkel, 2010). The impact of parental stress is not limited to parents' mental health but can also have significant consequences for children's development and well-being. Exposure to high levels of parental stress has been linked to a higher incidence of emotional and behavioral problems in children, such as anxiety, depression, and conduct disorders (Anthony et al., 2005; Cainelli et al., 2021). These effects can persist long-term, affecting children's academic and social development (Cheng & Lai, 2023).

High levels of parental stress have been linked to a variety of adverse outcomes in children, including behavioral problems, emotional dysregulation, and lower academic performance (Neece et al., 2012). Additionally, chronic stress in parents can affect parenting practices, leading to less responsive and more punitive interactions with their children (Crnic & Low, 2002). A determining factor in the occurrence and level of parental stress is parents' individual disposition to face the daily challenges of parenting. Parental stress is closely related to perceived parental competence, which directly influences the quality of parent–child interactions. Moreover, social support has been identified as a crucial moderator of parental stress; studies have shown that parents who perceive higher social support report lower levels of stress (Deater-Deckard, 1998). Socioeconomic and contextual variables' influence on parental stress has also been explored. In this regard, parents in poverty contexts face significantly higher levels of stress due to the combination of economic insecurity and limited access to support resources (Evans & Kim, 2013). Additionally, cultural differences can play an important role in how parental stress is perceived and managed, highlighting the need for context-sensitive research and practice approaches (Chao, 2001).

On the other hand, the COVID-19 pandemic exacerbated this situation due to lockdown measures and social restrictions, which increased caregiving responsibilities and limited access to support networks. This rise in responsibilities and lack of social support resulted in increased parental stress, negatively affecting the family environment (Jarvers et al., 2023). Recent studies indicate that this increase in stress was associated with a rise in problematic behaviors in children, both internal and external, as a result of accumulated tension and anxiety in the family environment (Geprägs et al., 2023). One study revealed that 71% of parents felt stressed when organizing their children's schooling (American Psychological Association (APA), 2020). The World Health Organization (WHO) emphasized that parenting was a significant challenge during the health crisis, causing concern and stress among parents due to the difficulty of talking to their children about COVID-19 and the need to create new learning routines at home to support education (Organización Mundial de la Salud (OMS), 2021).

The need for a measurement tool to promptly identify parental stress has led to the development of various instruments, with one of the most recognized being the Parenting Stress Index (PSI) initially developed by Loyd and Abidin. This inventory consists of 120 items distributed across three main stress domains: child characteristics, parent characteristics, and situational/demographic life stress (Loyd & Abidin, 1985). This instrument is used as a diagnostic or screening measure to evaluate the parenting system. Later, the instrument was shortened to the Parenting Stress Index-Short Form (PSI-SF), which consists of 36 items distributed across three subscales: 1) Parental Distress, 2) Parent–Child Dysfunctional Interaction, and 3) Difficult Child (Solis & Abidin, 1991). The first factor, Parental Distress (PD), refers to conflicts with the partner, the perception of lack of external support, and the responsibility to meet children's needs. The second factor, Parent–Child Dysfunctional Interaction, evaluates the conflictual relationship between parent and child, influenced by expectations and the quality of the relationship. The third factor, Difficult Child, refers to the child's characteristics, such as behavioral regulation, temperament, and behavioral problems perceived as unmanageable by the parents (Abidin, 1995; Solis & Abidin, 1991). Since Loyd and Abidin's (Loyd & Abidin, 1985) initial proposal to measure parental stress, the Parenting Stress Index-Short Form (PSI-SF) has been translated and validated in various languages (Aracena et al., 2016; Díaz-Herrero et al., 2010, 2011; Haskett et al., 2006; Lee et al., 2016; Luo et al., 2021; Rivas et al., 2020; Touchèque et al., 2016) (Table 1). Additionally, the PSI-SF has been applied to different population groups, such as parents with school-aged children (Haskett et al., 2006), parents of newborn babies (0–4 months) (Aracena et al., 2016), and infants aged 10 to 39 months (Díaz-Herrero et al., 2010, 2011), as well as Afro-descendant and Latino caregivers of children with behavioral difficulties (Lee et al., 2016).

**Table 1:** Psychometric Studies of the Parenting Stress Index Short Form (PSI-SF)

Authors and Year of Publication	Country	Sample size		#Of Factors	α
		**n**	**Population and Age Range**		
(Haskett et al., 2010)	United States	185	Parents: > 34 years, Children: 4–10 years	2	F1: .78 F2: .91
(Díaz-Herrero et al., 2010)	Spain	129	Mothers: 24–44 years, Children: 10–39 months	2	F1: .87 F2: .90
(Díaz-Herrero et al., 2011)	Spain	115	Parents: 25–49 years, Children: 10–39 months	3	F1: .84 F2: .82 F3: .82
(Lee et al., 2016)	United States	240	Black and Latino caregivers of children (7–11 years) with behavioral difficulties	3	F1: .89 F2: .82 F3: .83
(Aracena et al., 2016)	Chile	336	Mothers: 21 years, Children: < 4 months	3	F1: .81 F2: .89 F3: .88
(Touchèque et al., 2016)	France	210	Parents	3	F1: .81 F2: .79 F3: .79
(Luo et al., 2019)	China	683	Mothers: 28–62 years, Fathers: 29–69 years	3	Mother F1: .71 F2: .82 F3: .79 Father F1: .72 F2: .78 F3: .78
(Rivas et al., 2020)	Spain	309	Mothers with children aged 0–8 years	3	Alfa F1: .85 F2: .86 F3: .79 Omega F1: .86 F2: .86 F3: .79

Regarding the psychometric properties of the PSI-SF, several studies have confirmed its three-factor internal structure (Díaz-Herrero et al., 2011; Luo et al., 2021; Rivas et al., 2020; Touchèque et al., 2016). However, some studies suggest a two-factor structure (Díaz-Herrero et al., 2010; Haskett et al., 2006). In terms of reliability, few studies estimate the omega coefficient (Rivas et al., 2020), as most use the alpha coefficient, which is affected by the number of items and the ordinal nature of the responses (Cho, 2016). Therefore, it is important to conduct studies that provide a better understanding of the internal structure and reliability of the PSI-SF. Additionally, it is crucial to consider measurement invariance to ensure that members of different groups have the same understanding of the items on a scale. This helps verify whether individuals with the same level of a trait will respond similarly to the scale, regardless of their group membership (Milfont & Fischer, 2010). Without demonstrating invariance, conclusions derived from studies may be erroneous and biased towards one of the groups (Byrne, 2008; Dimitrov, 2010). It is worth noting that only one adaptation to the Spanish language in Latin America has been found (Aracena et al., 2016). Furthermore, no validation studies of this instrument have been found in the Peruvian context, nor are there valid comparisons between groups.

The objectives of this research were: a) To analyze the internal structure of the PSI-SF in a sample of parents using exploratory and confirmatory factor analysis; b) To estimate the reliability of the construct using the omega coefficient; c) To evaluate gender invariance.

## Methods

### Design and Participants

This is an instrumental study, as it analyzes the psychometric properties of a psychological measurement instrument (Ato et al., 2013). A total of 921 mothers and fathers with children aged 3 to 12 participated, with data collected in two phases. The first phase consisted of 130 participants for Exploratory Factor Analysis (EFA), and the second phase included 791 mothers and fathers for Confirmatory Factor Analysis (CFA). Both samples were obtained through intentional non-probabilistic sampling (Otzen & Manterola, 2017).

Table 2 details the sociodemographic characteristics of the study sample. The average age of the parents was higher (M = 37.0, SD = 7.1) in the exploratory analysis sample. The average number of children ranged between one and three. In both samples, the majority of participants were mothers, married, residing in the coastal region, and had children in primary education. Regarding occupation, in the EFA sample, 36.2% had an independent job, while in the CFA sample, 42.1% had a dependent job.

**Table 2:** Sociodemographic Characteristics of the Participants

Sociodemographic Variables	Exploratory Analysis (n = 130)		Confirmatory Analysis (n = 791)	
	M	D.E	M	D.E
Age of parents	37.0	7.1	34.4	6.8
Number of children	1.9	0.9	2.1	0.9
	n	%	n	%
Gender				
Male	27	20.8%	158	20.0%
Female	103	79.2%	633	80.0%
Residence				
Coast	113	86.9%	474	59.9%
Highlands	14	10.8%	183	23.1%
Jungle	3	2.3%	134	16.9%
Occupation				
Self-employed	47	36.2%	250	31.6%
Employee	39	30.0%	333	42.1%
Domestic activities	44	33.8%	208	26.3%
Marital status				
Married	72	55.3%	401	50.7%
Cohabiting	23	17.6%	258	32.6%
Divorced	7	5.3%	34	4.3%
Single parent	28	21.5%	98	12.4%
Child's education level				
Preschool	35	26.9%	168	21.2%
Primary	86	66.2%	413	52.2%
Preschool and primary	9	6.9%	210	266%

### Instrument

The instrument used for data collection was the Parenting Stress Index-Short Form (PSI-SF), designed by Abidin in 1995 (Abidin, 1995) and adapted into Spanish by Rivas et al. (Rivas et al., 2020). The scale consists of 36 items distributed across three subscales: 1) Parental Distress, 2) Parent–Child Dysfunctional Interaction, and 3) Difficult Child. Each subscale has 12 items with a Likert-type response format (1 = Strongly Disagree, 2 = Disagree, 3 = Not Sure, 4 = Agree, 5 = Strongly Agree), allowing scores ranging from 12 to 60. The total score is calculated by summing the scores of the three subscales, ranging from a minimum score of 36 to a maximum score of 180. Scores of 90 or above may indicate a clinical level of stress. The Spanish version with three factors, conducted by (Rivas et al., 2020), has reported adequate fit indices in a clinical sample (RMSEA = 0.06, CFI = 0.91, TLI = 0.90) and a non-clinical sample (RMSEA = 0.06, CFI = 0.92, TLI = 0.92) of mothers with children under eight years old. The alpha and omega coefficients range from 0.79 to 0.91, indicating adequate reliability.

### Procedure

Data collection was conducted in two phases. The first phase, for the exploratory factor analysis, took place in November and December 2021. The second phase, for the confirmatory factor analysis, was conducted from March to April 2022. Data were collected using virtual forms (Google Forms). The link to the form was shared through various communication channels and social media platforms such as Facebook, WhatsApp, email, phone calls, and video calls. The survey explained the research objectives and sought informed consent from each participant, stating that the information would be anonymous, confidential, and used solely for academic purposes. It was also mentioned that participation was voluntary and that participants could withdraw at any time if they wished. Each participant had the freedom to accept or decline to complete the questionnaire. Additionally, the email address of the researchers was provided for those participants who wanted more information about the research.

### Ethical Considerations

This study received approval from the ethics committee of the Peruvian Union University with approval reference number N° 2021-CE-FCS—UPeU-00318. The guidelines of the Declaration of Helsinki were also followed (World Medical Association (WMA), 2022).

### Data Analysis

Data analysis was conducted in four stages. In the first stage, content validation was performed through expert criteria, who reviewed the clarity, relevance, and representativeness of each item. A V-Aiken coefficient > 0.70 was considered indicative of content validity. In the second stage, exploratory factor analysis (EFA) was conducted to evaluate the theoretical and dimensional nature of the construct. The Kaiser–Meyer–Olkin (KMO) coefficient and Bartlett’s test of sphericity were used to meet the prerequisites. Parallel analysis (PA) was then used to determine the number of factors. For EFA, the unweighted least squares method with oblimin rotation was employed. In the third stage, confirmatory factor analysis (CFA) was conducted to analyze the internal structure of the scale. The robust weighted least squares estimator (WLSMV), which does not assume normality and is recommended for ordinal data, was used (Brown, 2015). To verify a good model fit, the indicators of the Comparative Fit Index (CFI), Tucker-Lewis Index (TLI), Goodness of Fit Index (GFI), and Adjusted Goodness of Fit Index (AGFI) were analyzed, all of which should be > 0.95 (Schermelleh-Engel et al., 2003; Schreiber et al., 2006). Additionally, the Root Mean Square Error of Approximation (RMSEA) and the Standardized Root Mean Square Residual (SRMR) were used, which should be < 0.06 or 0.08 to indicate an acceptable fit (Hu & Bentler, 1999; Mueller & Hancock, 2008; Schreiber et al., 2006). In the fourth stage, reliability was assessed using McDonald’s omega coefficient, with an adequate value being ω > 0.80 (Raykov & Hancock, 2005).

To ensure the factors of the instrument were consistent across genders, a series of increasingly stringent hierarchical variance models were applied. These models included first the configural invariance (reference model), followed by metric invariance (equal factor loadings), scalar invariance (equal factor loadings and intercepts), and finally strict invariance (equal factor loadings, intercepts, and residuals). A formal statistical test comparing the models was employed to assess the difference in the Comparative Fit Index (ΔCFI), where values below 0.010 indicated that the models were consistent across groups (Chen, 2007; Finch & French, 2018).

Statistical analyses were performed using R version 4.2.2 (R Project, 2022) and its free-access interface RStudio (RStudio, 2022). EFA and CFA were conducted with the Lavaan package version 0.6–8, and reliability was assessed with the SemTools package version 0.5–5.

## Results

### Content Validity

Before the expert review, some modifications were made to the parental stress scale to adapt it to the Peruvian context. The items adjusted were 3, 4, 5, 6, 10, 14, 16, 23, and 24. For example, item 3 in the Spanish version, which read "Me siento atrapado/a por mis responsabilidades como padre/madre" ("I feel trapped by my responsibilities as a parent"), was changed to "Siento presión por mis responsabilidades como padre/madre" ("I feel pressured by my responsibilities as a parent"). Similarly, item 23, which read "Esperaba tener más sentimientos de proximidad y calor con mi hijo/a de los que tengo, y eso me molesta" ("I expected to have more feelings of closeness and warmth with my child than I do, and it bothers me"), was modified to "Esperaba tener más sentimientos de proximidad y afecto con mi hijo/a de los que tengo, y eso me molesta" ("I expected to have more feelings of closeness and affection with my child than I do, and it bothers me"). Despite these modifications, the scale remained unaffected, as each expert rated the modified items positively, indicating better clarity and relevance in the scale.

Table 3 presents the Aiken's V coefficients, showing the content validation of the parental stress scale. Most items had coefficients above 0.70, indicating their suitability for the Peruvian context. However, items 6, 10, 11, 19, 22, 26, 29, and 30 had representativeness and relevance scores below 0.70, so they were reviewed and modified for application. For example, item 10, "Generalmente cuando voy a una fiesta no espero divertirme" ("Generally when I go to a party, I do not expect to have fun"), was changed to "Generalmente cuando voy a una reunión social no espero divertirme" ("Generally when I go to a social gathering, I do not expect to have fun"). Item 29, "Mi hijo/a reacciona muy fuertemente cuando sucede algo que no le gusta" ("My child reacts very strongly when something happens that they do not like"), was modified to "Mi hijo/a reacciona impulsivamente cuando le sucede algo que no le agrada" ("My child reacts impulsively when something happens that they do not like").

**Table 3:** Content Validity of the Parenting Stress Index Short Form (PSI-SF)

	Relevance	Representativeness	Clarity
Item 1	.75	.75	.83
Item 2	1.00	1.00	1.00
Item 3	1.00	1.00	1.00
Item 4	.92	.92	1.00
Item 5	1.00	1.00	1.00
Item 6	.50	.67	.83
Item 7	.83	.92	1.00
Item 8	1.00	1.00	1.00
Item 9	.83	.75	.92
Item 10	.67	.75	.83
Item 11	.75	.67	.92
Item 12	.83	.75	.83
Item 13	1.00	1.00	1.00
Item 14	1.00	1.00	.83
Item 15	.75	.75	1.00
Item 16	.75	.75	1.00
Item 17	.75	.75	1.00
Item 18	.92	.92	1.00
Item 19	.67	.67	.92
Item 20	.83	.83	1.00
Item 21	.92	.92	1.00
Item 22	.67	.58	.83
Item 23	.75	.75	1.00
Item 24	.75	.75	1.00
Item 25	.75	.75	1.00
Item 26	.67	.67	1.00
Item 27	.75	.75	1.00
Item 28	.92	.92	1.00
Item 29	.67	.67	.92
Item 30	.67	.67	1.00
Item 31	.92	.92	1.00
Item 32	1.00	1.00	1.00
Item 33	1.00	1.00	.92
Item 34	1.00	.92	.92
Item 35	1.00	1.00	1.00
Item 36	.92	.92	1.00

### Exploratory Factor Analysis

Before conducting the exploratory factor analysis (EFA), assumptions were verified. The Kaiser–Meyer–Olkin (KMO) coefficient was 0.89, which is considered high (Kaiser, 1974). Additionally, Bartlett’s test of sphericity showed appropriate values (χ^2^ = 137.78, df = 28, p = 0.001), demonstrating that EFA is suitable for exploring the dimensionality of the construct.

Using the Parallel Analysis (PA) method, the presence of three latent factors of the construct was suggested. EFA was then conducted to evaluate the internal structure of the scale, using the unweighted least squares method with oblimin rotation.

The EFA results show the presence of three latent factors (Parental Distress, Parent–Child Dysfunctional Interaction, and Difficult Child), which together explain 46.5% of the cumulative variance. Most items had factor loadings ranging from 0.315 to 0.868, exceeding the recommended minimum value of 0.30. However, items 8, 9, and 11, belonging to the "Parental Distress" factor, and items 21, 22, 25, 31, 26, 35, and 36, belonging to "Parent–Child Dysfunctional Interaction" and "Difficult Child," fit better with other theoretically unrelated factors, so they were discarded. Item 24 was included in the "Difficult Child" factor. In summary, three factors with 29 items and adequate factor loadings were identified, as shown in Table 4.

**Table 4:** Exploratory Factor Analysis with Oblique Rotation

Items	PD	PCHDI	DCH
E1	.481		
E2	.544		
E3	.678		
E4	.736		
E5	.868		
E6	.736		
E7	.596		
E10	.485		
E12	.595		
E13		.536	
E14		.727	
E15		.823	
E16		.702	
E17		.796	
E18		.478	
E19		.843	
E20		.512	
E23		.577	
E24			.594
E26		.487	
E27			.776
E28			.826
E29			.816
E30			.653
E32			.455
E33			.315
E34			.768
E35		.653	
E36		.487	

*PD* Parental Distress, *PCHDI* Parent–Child Dysfunctional Interaction, *DCH* Difficult Child. n = 130

### Confirmatory Factor Analysis

First, a descriptive analysis of the items was performed to examine the sample distribution, evaluating the mean, standard deviation, skewness, and kurtosis. Table 5 shows that item 3 had the highest mean score (M = 3.62; SD = 1.28), while item 26 had the lowest mean score (M = 2.90; SD = 1.37). Regarding skewness and kurtosis, the values did not exceed the recommended limits of ± 1.5 (Forero et al., 2009), indicating a normal distribution. Additionally, all items in the CFA had factor loadings ranging from 0.79 to 0.93, above the recommended minimum value of 0.30.

**Table 5:** Preliminary Analysis of Parenting Stress Index Short Form (PSI-SF) Items

Items	*M*	*DS*	*g*^*1*^	*g*^*2*^	*λ*
Parental Distress (PD)					
E1	3.59	1.22	-0.53	-0.78	.83
E2	3.56	1.25	-0.45	-0.97	.80
E3	3.62	1.28	-0.57	-0.87	.79
E4	3.34	1.34	-0.22	-1.25	.87
E5	3.36	1.34	-0.20	-1.28	.89
E6	3.27	1.38	-0.13	-1.35	.89
E7	3.36	1.30	-0.25	-1.18	.89
E8	3.23	1.38	-0.10	-1.36	.91
E9	3.36	1.32	-0.29	-1.20	.88
Parent–Child Dysfunctional Interaction (PCHDI)					
E10	3.08	1.43	0.01	-1.40	.92
E11	3.12	1.57	-0.28	-1.44	.92
E12	3.22	1.44	-0.22	-1.35	.86
E13	3.22	1.41	-0.12	-1.36	.91
E14	3.13	1.46	-0.03	-1.46	.92
E15	3.24	1.40	-0.18	-1.33	.91
E16	3.14	1.44	-0.06	-1.42	.92
E17	3.19	1.43	-0.11	-1.38	.92
E18	3.15	1.43	-0.08	-1.41	.93
E20	3.20	1.37	-0.06	-1.34	.90
E28	2.94	1.40	-0.02	-1.37	.91
E29	3.11	1.37	-0.17	-1.29	.90
Difficult Child (DCH)					
E19	3.33	1.33	-0.32	-1.16	.90
E21	3.40	1.32	-0.37	-1.09	.88
E22	3.53	1.21	-0.52	-0.77	.83
E23	3.39	1.28	-0.34	-1.08	.87
E24	3.40	1.28	-0.36	-1.04	.87
E25	3.56	1.21	-0.48	-0.75	.80
E26	2.90	1.37	-0.14	-1.39	.84
E27	3.31	1.20	-0.37	-0.94	.83

*M* Mean, *SD* Standard Deviation; g1 = Skewness; g2 = Kurtosis; λ = Factor Loading

Table 6 shows the CFA results for comparing the three proposed models. Model three demonstrated the best fit to the data, with adequate fit indices (GFI = 0.99, AGFI = 0.99, SRMR = 0.024, CFI = 0.98, TLI = 0.98, RMSEA = 0.074). The correlations between the three factors (Parental Distress, Parent–Child Dysfunctional Interaction, and Difficult Child) were 0.70 between PD and PCDI, 0.69 between PD and DC, and 0.78 between PCDI and DC, all significant. This indicates that the three factors present adequate discriminant validity.

**Table 6:** Confirmatory Factor Analysis of the Parenting Stress Index Short Form (PSI-SF)

	*X*^*2*^ (gl)	GFI	AGFI	SRMR	CFI	TLI	RMSEA [IC 90%]
Model 1 (One factor)	3441.89 (377) *p* < *.01*	.99	.99	.036	.97	.97	.101 [.098—.105]
Model 2 (Two factors)	2407.22 (376) *p* < *.01*	.99	.99	.032	.98	.98	.083 [.080—.086]
Model 3 (Three factors)	1993.39 (374) *p* < *.01*	.99	.99	.024	.98	.98	.074 [.071—.077]

χ2 = chi-square; *df* degrees of freedom, *GFI* Goodness of Fit Index, *AGFI* Adjusted Goodness of Fit Index, *SRMR* Standardized Root Mean Square Residual, *CFI* Comparative Fit Index, *TLI* Tucker-Lewis Index, *RMSEA* Root Mean Square Error of Approximation, *CI* Confidence Interval

Lastly, the internal structure of the scale, with the three factors and their respective items, was confirmed through CFA. The final scale consists of 29 items and demonstrates an adequate level of validity and reliability.

### Reliability

To assess the reliability of the scale, alpha and omega coefficients were used. The results indicate that the Parental Distress factor has an α = 0.94 and ω = 0.95; Parent–Child Dysfunctional Interaction has an α = 0.97 and ω = 0.97; and Difficult Child has an α = 0.94 and ω = 0.94. All factors exceed the recommended minimum value of 0.80 (Raykov & Hancock, 2005), suggesting that the scale is highly reliable.

### Factorial Invariance

Gender invariance was evaluated. Table 7 shows evidence of strict invariance according to the criterion (Δ CFI < 0.01). Adding the constraint of equal means did not significantly worsen the model fit, suggesting that the latent means are similar for both genders. Therefore, models M1, M2, M3, and M4 meet the expected criteria and confirm the factorial invariance of the PSI-SF. This allows for the comparison of different measures across gender groups.

**Table 7:** Factorial Invariance by Gender

	χ2	df	RMSEA	p	SRMR	TLI	CFI	∆CFI
M1	1126.4	748	.036	< .001	.029	.976	.978	
M2	1104.964	774	.033	< .001	.035	.980	.981	-0.003
M3	1140.396	800	.033	< .001	.036	.980	.980	0.001
M4	1164.385	829	.032	< .001	.036	.981	.981	-0.001

M1 = Configural; M2 = Metric; M3 = Scalar; M4 = Strict; χ2 = chi-square; *df* degrees of freedom; *RMSEA* Root Mean Square Error of Approximation, *SRMR* Standardized Root Mean Square Residual, *TLI* Tucker-Lewis Index, *CFI* Comparative Fit Index, *∆CFI* Comparative Fit Index difference

## Discussion

Parental stress has become a daily issue within households due to the high levels of stress parents experience while caring for their children and managing additional work responsibilities (Araya, 2021). Many have tried to balance their personal lives, work, and parenting but have found themselves without resources or support to cope with the stress (Spinelli et al., 2020). On the other hand, increased parental stress negatively impacts children's development and adjustment, leading to emotional and behavioral problems (Betancourt-Ocampo et al., 2021; Fang et al., 2024), sleep disturbances, and cognitive and behavioral disorders (Okelo et al., 2024; Orgilés et al., 2023). Additionally, it contributes to social adaptation issues and mental health problems in adolescents (Guo et al., 2024). Therefore, the aim of this study was to analyze the factorial structure of the Spanish version of the Parenting Stress Index-Short Form (PSI-SF) in parents with children aged 3 to 12 years.

First, the results of the Exploratory Factor Analysis (EFA) indicated the presence of three latent factors related to the construct. However, some items (8, 9, 11, 21, 22, 25, and 31) had low loadings and grouped in a theoretically inappropriate factor, distorting Abidin's original proposal (Abidin, 1995). Therefore, they were removed from the construct. Additionally, some items grouped into different factors than originally proposed, requiring conceptual analysis and decisions about their placement. For instance, item 24, "Sometimes my child does things that bother me just for the sake of doing them," initially proposed for the PCDI factor, was moved to the DC factor. Similarly, item 26, "My child usually wakes up in a bad mood," item 35, "My child has become a bigger problem than I expected," and item 36, "My child demands more from me than most children," originally in the DC factor, were moved to the PCDI factor. Consequently, the Spanish version of the Parenting Stress Index-Short Form (PSI-SF) preliminarily consisted of 29 items, distributed as 9 items for the PD factor, 12 items for the PCDI factor, and 8 items for the DC factor.

Subsequently, CFA was conducted on the modified version of the Parenting Stress Index-Short Form (PSI-SF). Three internal structure models proposed by Reitman et al. (Reitman et al., 2002) were analyzed. Our results demonstrated that model three showed the best fit indices, suggesting that a three-factor latent structure is most appropriate for the scale. This finding differs from the results of Haskett et al. and Díaz-Herrero et al. (Díaz-Herrero et al., 2010; Haskett et al., 2006), who reported a two-factor structure. However, more recent studies have supported a three-factor latent structure (Aracena et al., 2016; Lee et al., 2016; Luo et al., 2021; Rivas et al., 2020; Touchèque et al., 2016), consistent with previous research and Abidin's original proposal (Abidin, 1995). Additionally, support for the three-factor model derives from its greater clinical utility (Abidin, 1995).

The three latent factors identified are as follows: The first factor, Parental Distress (PD), refers to conflicts with the partner, disagreements in parenting styles, lack of external support for child care, and the perceived responsibility parents have to meet their children's needs. The second factor, Parent–Child Dysfunctional Interaction (PCDI), evaluates the conflictual relationship between parent and child, generally related to parents' expectations of their children, the quality of their relationship, and the reinforcement they receive from their children. The third factor, Difficult Child (DC), pertains to the child's characteristics, which parents perceive as easy or difficult in terms of behavior regulation, temperament perception, behavioral problems, emotional regulation, or any condition perceived as unmanageable by the parent.

Regarding the internal consistency of the PSI-SF, most previous studies have used the alpha coefficient to analyze the scale's reliability (Aracena et al., 2016; Díaz-Herrero et al., 2010, 2011; Haskett et al., 2006; Lee et al., 2016; Luo et al., 2021; Touchèque et al., 2016). However, the alpha coefficient is affected by the number of items and the ordinal nature of responses (Cho, 2016; Domínguez-Lara & Merino-Soto, 2015). In contrast, the omega coefficient works with factor loadings, making the calculation more stable and reporting reliability more accurately. This study estimated reliability using the omega coefficient, and all scale factors obtained coefficients above 0.80 (Raykov & Hancock, 2005), indicating high reliability of the PSI-SF.

Factorial invariance analysis concerning gender demonstrated that thresholds, factor loadings, intercepts, and residuals remained stable across groups, indicating that the items measure the latent variable in the same way for both men and women (Brown, 2015). Therefore, it can be affirmed that differences in scores between men and women are due to differences in the latent trait and not to bias in the measurement instrument. These results are important as they will enable future studies on self-efficacy based on gender and provide useful information for its application.

The findings of this study suggest several implications regarding parental stress. The PSI-SF is a valuable tool that allows for the assessment and quantification of the level of stress that parents experience in their parental role. Mental health professionals and educators will not only be able to identify the implications of this detrimental phenomenon but also intervene in a timely and effective manner. It is recommended that therapists and educators incorporate strategies focused on training to manage negative emotions such as anger and frustration. These skills could help parents strengthen their self-regulation abilities in their parenting practices. Additionally, educational institutions could consider establishing interdisciplinary programs aimed at reducing parental stress and improving family well-being, promoting positive parent–child interactions, and enhancing the functional role of parents, with the objective of improving the quality of family dynamics.

Moreover, these findings could be useful for the implementation of new labor policies and/or social services that recognize and address the challenges related to fulfilling the parental role that workers face when they become parents and the changes and responsibilities that this entails. This would help protect the mental health of parents without affecting job performance. Lastly, future research could explore the relationship between parental stress and other psychological constructs to understand the underlying mechanisms of family dynamics and the role of stress in parents' lives. Conducting longitudinal and experimental studies could provide a deeper understanding of these relationships and help elucidate causal pathways. Additionally, considering a more diverse population with parents from different educational and cultural backgrounds could provide a better understanding of the phenomenon of parental stress in various contexts.

The findings of this study should be considered in light of some limitations. One limitation is the discrepancy between the samples of the EFA and CFA, as there is a disproportion among the categories of variables. The use of non-probability sampling increases the likelihood of selection bias, which prevents the generalization of findings to the entire Peruvian population. Most participants were women, lived in the coastal region, were married, and had children in primary education. Although it is important to consider the size and proportionality of categories, it is not an absolute requirement for conducting a factorial invariance analysis regarding the gender of parents. Therefore, it is recommended that future studies employ representative samples using probabilistic sampling to generalize the results. Additionally, the absence of information on the educational level of parents is another limitation of the study. The use of self-report measures could predispose participants to social desirability bias and generate response biases, so it is recommended to complement the research with semi-structured interviews in future studies. Another limitation is the lack of concurrent validity, so it is suggested to use variables related to the construct as evidence of the concurrent validity of the scale.

## Conclusion

Despite these limitations, one of the strengths of this study is that it provides empirical evidence for a new version of the Parenting Stress Index-Short Form (PSI-SF), comprising 29 items that support the three-factor latent model of the scale. This is significant as there is only one adaptation of this questionnaire to American Spanish, and it has not been validated for Peruvian parents. Therefore, this study provides evidence of adequate validity and reliability for its use with parents of children aged 3 to 12 in the Peruvian population. Another strength of the study is the inclusion of a factorial invariance analysis based on the gender of the parents, which was absent in previous studies. This is crucial to ensure that the instrument is invariant and that the observed differences between fathers and mothers are genuine and not due to measurement bias.

## Data Availability

The datasets used and/or analyzed during the current study are available from the corresponding author on reasonable request.

## Abbreviations

PSI-SF: Parenting Stress Index Short Form
EFA: Exploratory factor analysis
CFA: Confirmatory factor analysis
KMO: Kaiser–Meyer–Olkin
PA: Parallel analysis
WLSMV: Weighted Least Squares Mean and Variance Adjusted
CFI: Comparative fit index
TLI: Tucker-Lewis index
GFI: Goodness of fit index
AGFI: Adjusted goodness of fit index
RMSEA: Root mean square error of approximation
SRMR: Standardized root mean square residual
